# More Power, More Warmth: The Enhancing Effect of Power on the Perceived Warmth About High-Power Individuals Under Chinese Culture

**DOI:** 10.3389/fpsyg.2022.874861

**Published:** 2022-06-02

**Authors:** Minyan Li, Feng Yang, Yang Han

**Affiliations:** Teacher Education Department, Taishan University, Taian, China

**Keywords:** power, stereotypes, Confucianism, stereotype content model, social cognition, impression formation

## Abstract

Previous literature concerning power stereotypes demonstrates that compared to low-power (LP) individuals, high-power (HP) individuals tend to be perceived as having positive competence but negative warmth. Based on previous research, the current research further classified HP into senior and junior HP and mainly compared the perceived warmth between senior and junior HP individuals in Chinese culture. By classifying power into HP and LP, the pilot study employed the trait-rating task to replicate the results of previous research. In Study 1, we classified HP into senior and junior HP and revealed that participants indicated more positive warmth evaluations for senior HP individuals than for junior HP individuals. We named this “more power, more warmth” effect the MPMW effect. Further investigation demonstrated that the MPMW effect was more likely to emerge for participants with high Confucianism identification (Study 2a), for Chinese participants rather than Western participants (Study 2b), or when the knowledge of Confucianism was accessible in a given situation (Study 3). The present research firstly demonstrated that the contents of power stereotypes may partially display culture-specific characteristics in Chinese culture. The continuous classification approach to power provided a novel insight for future power research.

## Introduction

In China, there is a well-known proverb “It's easy to deal with Yama, but hard to do with devilkins”. Under Chinese culture, the “devilkins” generally refer to those junior HP individuals who are primarily responsible for directly managing ordinary people in a society or employees in a company, such as community directors, workshop managers, etc. The “Yama” often refers to those senior HP individuals who are primarily responsible for managing junior HP individuals rather than ordinary people in a society or employees in a company, such as ministers, Board Chairman, etc. From the perspective of social psychology, the proverb reflects the consensus of Chinese people about power stereotypes. That is, if HP individuals are divided into senior HP individuals (e.g., Board Chairman) and junior HP individuals (e.g., Workshop Manager), compared to junior HP individuals, senior HP individuals will be considered to display more positive warmth traits in communication with others.

It should be pointed out, to our knowledge, that previous power literature commonly investigates the effect of power by simply classifying power into HP and LP (Anicich et al., 2020), so we are not sure whether the proverb mentioned in the beginning can receive the supports from empirical research. In other words, although previous power stereotype research in Chinese culture has consistently demonstrated that more power is generally associated with less perceived warmth when comparing HP with LP individuals (Zhang et al., 2015; Wang et al., 2017), it is still unclear whether the elevation of power will be associated with more/less perceived warmth about senior and junior HP individuals. To fill this gap, we conducted four studies on Chinese culture to examine whether there was the “more power, more warmth (MPMW)” effect—more power was associated with more warmth perception about the stereotypes of HP individuals, and we also attempted to specify the condition for the emergence of the MPMW effect. It should be noted that the so-called “more power, more warmth” effect in the research exclusively referred to the warmth perception comparison between senior and junior HP individuals, rather than the warmth perception comparison between HP (including senior and junior HP) and LP individuals.

## Literature Review

### Stereotype Content Model and Power Stereotypes

In order to make sense of our surrounding social world without being cognitively overwhelmed, we humans tend to process information about others based on their social categories (Fiske and Neuberg, 1990; Carlsson and Björklund, 2010). We do this by employing stereotypes, which are considered to be some socially shared beliefs that the members of a social category commonly possess some typical traits or characteristics (Hilton and Hippel, 1996). To date, researchers have revealed various stereotypes, such as race, gender, and age stereotypes (Devine, 1989; Banaji and Hardin, 1996; Chasteen et al., 2002). An interesting phenomenon pertaining to stereotypes is that the stereotypes of a specific group often contain a mix of traits that widely differ in evaluative orientation (Fiske et al., 2002; Cuddy et al., 2008). For example, stereotypes about lawyers include some typically positive traits (e.g., smart, professional) and at the same time include some typically negative traits (e.g., pitiless, profit-oriented). In order to describe this mixed content of stereotypes, Fiske et al. (2002) developed the Stereotype Content Model (SCM). According to the SCM, warmth, and competence are universal and fundamental dimensions in social judgments and warmth is the primary dimension (Fiske et al., 2007; Cuddy et al., 2008). The warmth dimension mainly answers the question regarding whether the other person is friendly or hostile in social interaction, and the competence dimension represents the person's capability to carry out the intention (Fiske et al., 2007). The four combinations of high vs. low warmth and competence elicit four unique patterns of behavioral and emotional responses (for a detailed review, see Cuddy et al., 2008). The SCM further postulates that people often hold ambivalent stereotypes toward out-group members—they tend to be evaluated as positive on one dimension and negative on the other dimension. For instance, old people are stereotyped as warm but incompetent and lawyers are perceived as highly competent but cold (Carlsson and Björklund, 2010). Supporting the proposition of ambivalent stereotypes, past research in the power field suggests that HP individuals tend to be stereotyped as having positive competence but negative warmth. That is, they commonly receive positive evaluations on the competence dimension but negative evaluations on the warmth dimension. In contrast, LP individuals tend to be stereotyped as having negative competence but with positive warmth. That is, they commonly receive negative evaluations on the competence dimension but positive evaluations on the warmth dimension (Russell and Fiske, 2008; Fragale et al., 2009; Zhang et al., 2013, 2015).

Power is a fundamental part of our social life and has profound effects on our psychological and behavioral processes (Fiske and Dépret, 1996; Magee and Galinsky, 2008). Generally, power is defined as an individual's relative capacity to modify others' states by providing/withholding resources or administering punishments (Keltner et al., 2003). In essence, power means asymmetric control or dependence—LP individuals depend on HP individuals but HP individuals are independent of LP individuals (Lammers et al., 2012). Thus, HP individuals are less motivated to pay attention to LP individuals surrounding them, because they can achieve their goals without the assistance of LP individuals (Chen et al., 2009; Guinote et al., 2012). In contrast, LP individuals need to display socially desirable traits so that they can acquire necessary resources from HP individuals (Magee and Smith, 2013). As a result, in line with the propositions of the SCM, HP individuals are inclined to be perceived as positive competent but negative warmth, and LP individuals are inclined to be perceived as negative competent but positive warmth. For an instance, Zhang et al. (2015) utilized the implicit association test (IAT) to detect the contents of power stereotypes. With regard to the target dimension, they selected 16 power labels as target words: 8 HP labels and 8 LP labels. With regard to the warmth dimension, they selected 16 power-stereotype-relevant traits as attribute words: 8 HP stereotype-consistent traits (4 positive competence traits and 4 negative warmth traits) and 8 LP stereotype-consistent traits (4 negative competence traits and 4 positive warmth traits). Following the standard procedure of the classic IAT (Greenwald et al., 1998), participants needed to respond to the compatible combinations (HP–positive competence/negative warmth, LP–negative competence/positive warmth) in Parts 3 and 4, and respond to the incompatible combinations (HP–negative competence/positive warmth, LP–positive competence/negative warmth) in Part 6 and 7. The results showed that participants generally needed longer reaction times to respond to the incompatible combinations than the compatible combinations, resulting in a significantly greater D value than 0. The results of the research suggested that participants tended to perceive HP individuals as positive competent but negative warmth and perceive LP individuals as negative competent but positive warmth.

### Power Construction and Confucianism

In the Han dynasty of China, the emperor Wudi established Confucianism as the only accredited philosophy in order to maintain social stability—the so-called “Ban from hundred philosophers, venerate Confucianism”. Since then, Confucianism has been the dominant philosophy in China for nearly two thousand years and produces a far-reaching influence on the Chinese people (Fung, 1948/2018). Regarding how one person should exert his power, Confucianism on the hand asserts that power-holders should have a great social responsibility and devote their lives to serving the whole society (Chen and Chung, 1994; Hwang, 2012). For example, Confucianism declares that an excellent governor should love ordinary people as if they were his own children. In line with this declaration, empirical research also suggests that HP individuals under Confucian culture are expected to have great social responsibility and concern for the wellbeing of others (Low and Ang, 2012, 2013). In this sense, HP individuals are expected to display positive warmth in communication with others. However, on the other hand, Confucianism puts an emphasis on the stateliness, status, and even privilege of HP individuals in daily communications (Chen and Chung, 1994; Chen et al., 2013). For instance, Chen et al. (2013) found that under Confucian culture, when a leader put forward a project plan, those subordinates tended to express advocacy toward the plan rather than directly raised doubts or modifications, because the latter could be regarded as a challenge toward the authority of power. As a result, in daily communications with others, HP individuals may be perceived as negative warmth due to the emphasis on the hierarchical order. Taken together, we reasoned that Confucianism may lead to being the discrepancy in the impressions between the expected HP individuals in the public mind and the encountered HP individuals in real communications—HP individuals tend to be associated with positive warmth in expectation but tend to be associated with negative warmth in real communications.

Although the above proposition to date has received less empirical support, to some extent, it is consistent with the widely accepted individualism/collectivism classification hypothesis, which defines Chinese society as a vertical collectivist society (VC; Singelis et al., 1995; Triandis, 1995; Triandis and Gelfand, 1998; Triandis et al., 1998). Specifically, Torelli and Shavitt (2011) have pointed out that individuals in the VC society have relatively complex conceptualizations of power. On one hand, HP individuals in the VC society are considered to put an emphasis on personal success and status in the hierarchy, and subordinates' compliance to the will of authority, which may prompt them to display negative warmth in communication with others. Meanwhile, HP individuals in the VC society are expected to join others in the group to compete with out-groups, and even sacrifice personal benefits for the sake of in-group goals. In this sense, they seemingly are expected to be positive on the warmth dimension. To some extent, these seemingly contradictory characteristics displayed by HP individuals in the VC society are consistent with our proposition about the discrepancy between the expected HP individuals and the perceived HP individuals in real experiences. Moreover, beyond previous research adopting the traditional individualism/collectivism perspective, our Confucian perspective provided an alternatively deeper explanation for the seemingly contradictory characteristics of HP individuals in the VC society—it may root in Confucianism.

One may question if the discrepancy exists between the expected HP individuals and the perceived HP individuals in real experiences, why previous research on Chinese culture consistently demonstrates that HP individuals are perceived to be positive competence but negative warmth (Zhang et al., 2013, 2015; Wang et al., 2017). It should be pointed out, that previous research in the power field commonly classifies power into HP and LP in a dualistic way, no research to date has divided HP into senior HP and junior HP (Anicich et al., 2020). However, for ordinary Chinese people, although junior HP individuals (e.g., local officials) and senior HP individuals (e.g., central officials) both belong to HP individuals, they actually may have different impressions about such two types of HP individuals. One important reason for our reasoning is that in daily life, most ordinary people interact with junior HP individuals rather than senior HP individuals. So, for most people, the accessibility of the knowledge of junior HP individuals should be higher than the accessibility of the knowledge of senior HP individuals. Accessibility generally refers to a state that is produced by prior processing of a stimulus and thus activates knowledge (Förster and Liberman, 2007). In this case, when mentioning HP individuals by default, most people may form the impression about them based on their experiences of interacting with junior HP individuals, and then utilized the impressions of junior HP individuals to represent the whole HP individuals. So, our first hypothesis was:

*When power was divided into high power and low power, consistent with previous theoretical and empirical research, individuals in Chinese culture would perceive HP individuals as positive competence but negative warmth* (Hypothesis 1).

According to our thesis 1, when HP individuals have not been divided into senior and junior HP individuals, Chinese people may (unconsciously) rely on their experiences of interacting with junior HP individuals to form an impression about the whole HP individuals. As a result, HP individuals tend to be perceived as having negative warmth. However, upon explicitly making a distinction between senior and junior HP individuals, people still can form the impression about junior HP individuals based on their real experiences, whereas such experiences are less available to represent senior HP individuals. In this case, as a chronically accessible construct (for a detailed introduction to the accessibility theory, see Higgins, 2012), Confucian constructions about HP individuals thus can be easily retrieved and used in impression formation about junior HP individuals. Indeed, past stereotype literature has suggested that some task-relevant information with the ease of retrieval tends to be used in impression formation with a high priority (Gawronski and Bodenhausen, 2005; Weick and Guinote, 2008). Considering that Confucianism alleges that HP individuals should have great social responsibility and concern for others, our second hypothesis was:

*When explicitly classifying HP individuals into senior and junior HP individuals, participants may give more positive warmth evaluations for senior HP individuals than for junior HP individuals* (the MPMW effect, Hypothesis 2).

According to our previous reasoning, due to the dominance of Confucianism in China, Chinese people may give more positive evaluations on the warmth dimension for senior HP individuals than for junior HP individuals. Following this reasoning, we can further speculate that if individuals in a specific situation have less identification with Confucianism, they should be less likely to evaluate senior HP individuals as positive warmth. In other words, individuals' identification with Confucianism may moderate the MPMW effect—in a specific situation, only when the majority of individuals keep identification with Confucianism, can our expected MPMW effect on HP individuals appear. As for the competence dimension, considering that the social responsibility emphasized by Confucianism mainly has an influence on the warmth perceptions of senior HP individuals, we expected that the competence perceptions toward senior HP individuals were insensitive to participants' Confucianism identification. So, our third hypothesis was:

*Participants' identification with Confucianism was the premise of the MPMW effect: Only when most participants keep identification with Confucianism in a specific situation, would they give more positive warmth evaluations for senior HP individuals than for junior HP individuals* (Hypothesis 3).

In addition to the above three issues, out of curiosity, we also wanted to know whether Chinese participants would give more positive warmth evaluations for senior HP individuals than for LP individuals. However, because of lacking relevant literature, we cannot make a clear assumption about the warmth comparison between senior HP individuals and LP individuals. Moreover, this is not our major concern in the current research. Given that, we would conduct an exploratory investigation about this issue.

## The Current Research

Overall, the current research aimed to explore the “more power, more warmth (MPMW)” effect of HP stereotypes in Chinese culture, and the determining role of Confucianism in the existence of the MPMW effect. To this end, we firstly conducted a pilot study in which we used the trait-rating task to preliminarily examine the contents of power stereotypes about HP and LP individuals. We anticipated that consistent with previous research (Russell and Fiske, 2008; Fragale et al., 2009; Zhang et al., 2013), HP individuals tended to be stereotyped as positive competence but negative warmth while LP individuals tended to be stereotyped as negative competence but positive warmth. Based on this, we then conducted Study 1 in which we again used the trait-rating task to examine the contents of power stereotypes about senior HP, junior HP, and LP individuals. In Study 1, we put our main focus on the comparison between senior and junior HP stereotypes, and we predicted that compared to junior HP individuals, senior HP individuals tend to be perceived as more positive on both competence and warmth dimensions. In the following Study 2a and 2b, by using the trait-rating task, we exclusively compared the stereotypes about senior and junior HP individuals and also explored the role of Confucianism in the MPMW effect. In Study 2a, we developed the Confucianism identification scale to examine whether the MPMW effect only emerged for participants displaying high identification with Confucianism. In Study 2b, we conducted an online cross-cultural study (Chinese participants vs. Western participants) to examine whether the MPMW effect appeared among Chinese participants rather than Western participants. In Study 3, we presented the counter-Confucianism stimuli to temporarily reduce the accessibility of Confucianism and then compared the magnitude of the MPMW effect in the implicit association task (IAT) between the Counter-Confucianism and the control conditions (no presenting the counter-Confucianism stimuli). We predicted that, due to the temporary decline of the Confucianism accessibility under the Counter-Confucianism condition, the MPMW effect would be significantly attenuated under this condition.

## Pilot Study

Before examining power stereotypes in a relatively continuous way, we firstly conducted a pilot study in which we classified power into HP and LP in a dualistic way and investigated the contents of power stereotypes via the trait-rating task. By doing so, we not only could give the empirical test for our hypothesis 1 but also could establish the basis for our further investigation of HP stereotypes. In the trait-rating task, we selected “leader” and “ordinary people” as two target groups, and for each target group, participants needed to indicate to what extent the given traits (including positive and negative traits on both competence and warmth dimensions) were suitable to describe that group. To confirm the fitness between the selected traits and the two presupposed dimensions (competence and warmth), in another independent sample (*n* = 46, 18 men, 18 women, *M*_age_ = 20.64, *SD* = 3.14), we asked participants to report whether and to what extent each pair of traits belong to the competence or the warmth dimension on an 11-point scale (ranging from −5 to 5, −5 = completely belong to the competence dimension, 5 = completely belong to the warmth dimension). Then, following previous research (Leach et al., 2007), a confirm factor analysis (CFA) was performed on participants' scores with a maximum likelihood estimation. In the CFA, the two latent factors were allowed to correlate, but no errors on specific items were allowed to correlate. The results showed an acceptable mode fitness, χ^2^*/df* = 1.17, *p* = 0.23, *CFI* = 0.93, *TLI* = 0.91, *SRMR* = 0.08, *RMSEA* = 0.06 (Hu and Bentler, 1999).

According to prior literature (Russell and Fiske, 2008; Fragale et al., 2009; Yang et al., 2019), in the trait-rating task of the pilot study, we speculated that participants would rate HP individuals as positive competence but negative warmth, and LP individuals as negative competence but positive warmth. More detailed introductions about the trait-rating task would be provided in Section Materials and Procedure.

### Participants and Design

A pilot study was a 2 (target power: high vs. low) × 2 (stereotype dimension: competence vs. warmth) within-subjects design. According to the calculation of the G^*^Power 3.1 (https://www.psycho.uni-duesseldorf.de/abteilungen/aap/gpower3 Faul et al., 2009; a presupposed medium effect size of 0.25 and being significant at the 0.05 level), on a voluntary basis, we recruited 40 undergraduates to participate in the study (16 men, 24 women, *M*_age_ = 21.66 years old, ranging from 20.92 to 23.08). All participants were assigned the informed content before starting the task. For their participation, they can choose to get 10 RMB cash or a small gift worth 10 RMB.

### Materials and Procedure

Participants participated in the pilot study in a group of 6–8. Upon arriving at the lab, they were told that they would take part in an impression formation task, and if they agreed, they needed to assign the informed content. After that, we introduced the trait-rating task for participants.

The trait-rating task consisted of two targets needing to be evaluated—*leaders* and *ordinary people*. In Chinese culture, “leader” refers to those HP individuals in a general way, and “ordinary people” refers to those LP individuals in a general way. Besides such two evaluated targets, the task included 10 pairs of traits, which were selected from previous research (Zhang et al., 2013; Wang et al., 2017). Such 10 pairs of traits included 5 pairs of competence traits and 5 pairs of warmth traits, and each pair consisted of two traits with opposite meanings (e.g., smart vs. muddleheaded). These traits were listed in Table 1. For each target, participants needed to mark a number on an 11-point scale (ranging from −5 to 5) to indicate which of each pair was more suitable to describe the target, and to what extent it was suitable to describe the target. Specifically, if participants marked a negative number on the scale, it means that the negative trait was considered to be more suitable to describe the target group and vice versa. Under this logic, “−5” implied that the negative trait was very suitable for the target group, and “5” implied that the oppositely positive trait was very suitable for the target group. In this way, for each target, participants were asked to give their ratings across all 10 pairs of traits. The presentation order of the targets was counterbalanced across participants.

**Table 1:** Ten pairs of traits in the trait-rating task of the pilot study.

	**Positive**	**Negative**
Competence traits	Smart	Muddleheaded
	Vigilant	Numb
	Self-confident	Self-contemptuous
	Resourceful	Helpless
	Indecisive	Decisive
Warmth traits	Friendly	Unfriendly
	Upright	Cunning
	Genuine	Hypocritical
	Enthusiastic	Indifferent
	Modest	Supercilious

After completing the trait-rating task, participants needed to provide necessary demographic information. Then, we expressed our thanks to them and answered their questions about the study. Finally, we gave each participant a small gift or 10 RMB cash (following their preference) and guided them to leave the lab.

### Results

For each participant, we calculated his/her rating scores toward each target by separately averaging the scores on the competence and warmth dimensions. Thus, four types of scores were generated for each participant: HP-competence, HP-warmth, LP-competence, and LP-warmth. Preliminary analyses did not reveal any significant main effect of gender or interaction effect with other variables, so we dropped this variable from formal data analyses. To assess how the ratings of targets varied with target power and stereotype dimension, we conducted a 2 (target power: high vs. low) × 2 (stereotype dimension: competence vs. warmth) within-subjects ANOVA. Mean (*M*) and standard deviation (*SD*) in each condition were presented in Table 2. The results of this ANOVA analysis showed a significant main effect of target power, *F*_(1, 39)_ = 4.71, *p* = 0.04, partial η^2^ = 0.11, with higher score for the HP target than for the LP target (*M* = 1.63, 1.27, respectively). In addition, the main effect of stereotype dimension also was significant, *F*_(1, 39)_ = 24.58, *p* < 0.001, partial η^2^ = 0.39, with higher score on the competence dimension than on the warmth dimension (*M* = 1.98, 0.92, respectively). However, the above significant main effects were qualified by a significant interaction between target power and stereotype dimension, *F*_(1, 39)_ = 99.87, *p* < 0.001, partial η^2^ = 0.72. Further simple effect analyses showed that participants indicated a higher score on the competence dimension for the HP target than for the LP target, *t*_(39)_ = 11.22, *p* < 0.001, d = 2.6. By contrast, on the warmth dimension, participants indicated a higher score for the LP target than for the HP target, *t*_(39)_ = 6.64, *p* < 0.001, d = 1.29. Simple effect analyses from the other direction showed that participants indicated a higher score on the competence dimension than on the warmth dimension for the HP target, *t*_(39)_ = 8.71, *p* < 0.001, d = 2.18, and showed the opposite results pattern for the LP target, *t*_(39)_ = 7.46, *p* < 0.001, d = 1.25.

**Table 2:** Participants' average rating score in each condition of the pilot study (*M* ± *SD*).

	**HP target**	**LP target**
Competence	3.55 ± 0.87	0.42 ± 1.46
Warmth	−0.28 ± 2.33	2.12 ± 1.24

*N = 40. HP, high power; LP, low power*.

### Discussion

Supporting our hypothesis 1, the results of the pilot study demonstrated that participants tended to evaluate HP individuals as highly competent but with low warmth, and evaluate LP individuals as low competence but high warmth. The pilot study not only conceptually replicated previous power stereotype literature (Russell and Fiske, 2008; Fragale et al., 2009; Zhang et al., 2013), but also provided a theoretical and statistical basis for our further investigation of power stereotypes. In the trait-rating task of the following Study 1, target power would be subdivided into senior HP, junior HP, and LP, so that we can assess whether there would be significant differences in the stereotypes between senior and junior HP individuals.

## Study 1

In the above pilot study, we employed the trait-rating task to assess the stereotypes about HP and LP individuals. Consistent with our hypothesis 1, the results demonstrated that participants tended to rate HP individuals as positive competence but negative warmth, and rate LP individuals as negative competence but positive warmth. Based on this, Study 2 further classified the rated targets into senior HP, junior HP, and LP targets and examined the power stereotypes in a more subtle way. More concretely, our major concern in Study 1 was whether there were any differences in the stereotypes between senior and junior HP individuals. According to our hypothesis 2, we speculated that compared to junior HP individuals, senior HP individuals tended to be perceived as more positive warmth. It should be explained in advance that although our core concern in Study 1 was the warmth comparison between senior and junior HP targets, to avoid disrupting participants' perceived integrity and comprehensiveness about the trait-rating task, participants in Study 1 still needed to give their evaluations about targets on both competence and warmth dimensions.

### Participants and Design

Study 1 was a 3 (target power: senior HP vs. junior HP vs. LP) × 2 (stereotype dimensions: competence vs. warmth) within-subjects design. According to the calculation of G^*^Power 3.1 (Faul et al., 2009), at least 28 participants can meet the criterion of a medium effect size of 0.25 and significance at a 0.05 level. Considering possible invalid data, we finally recruited 32 undergraduates to participate in the study. All participants were assigned the informed content before the formal task. For their participation, participants can get 10 RMB cash or a small gift worth 10 RMB.

### Materials and Procedure

This section was identical to that of the pilot study except that participants needed to give their ratings for three types of targets in the trait-rating task—*senior leader, junior leader*, and *ordinary people*. We told participants that, the “senior leader” generally refers to those senior HP individuals who are primarily responsible for managing junior HP individuals rather than ordinary people in a society or employees in a company. And the “junior leader” generally refers to junior HP individuals who are primarily responsible for managing ordinary people in a society or employees in a company. As for the “ordinary people”, they generally refer to those people who have little power and are managed by HP individuals. Similar to the pilot study, for each target, participants needed to mark a number on an 11-point scale (ranging from −5 to 5) to indicate which of each pair was more suitable to describe the target, and to what extent it was suitable to describe the target. Again, the presentation order of the targets was counterbalanced across participants.

After the trait-rating task, participants needed to provide necessary demographic information. Then, we expressed our thanks to them and answered their questions about the study. Finally, they got a small gift or 10 RMB cash (following their preference) and left the lab.

### Results

As did in the pilot study, we calculated each participant's rating scores toward each target by separately averaging the scores on the competence and warmth dimensions. The average score of all participants in each condition was presented in Table 3.

**Table 3:** Participants' average rating score in each condition of Study 1 (*M* ± *SD*).

	**Senior HP target**	**Junior HP target**	**LP target**
Competence	3.32 ± 1.37	1.51 ± 1.52	−0.76 ± 1.54
Warmth	0.67 ± 2.36	−1.43 ± 1.60	1.58 ± 1.65

*N =32. HP, high power; LP, low power*.

**Figure 1:**
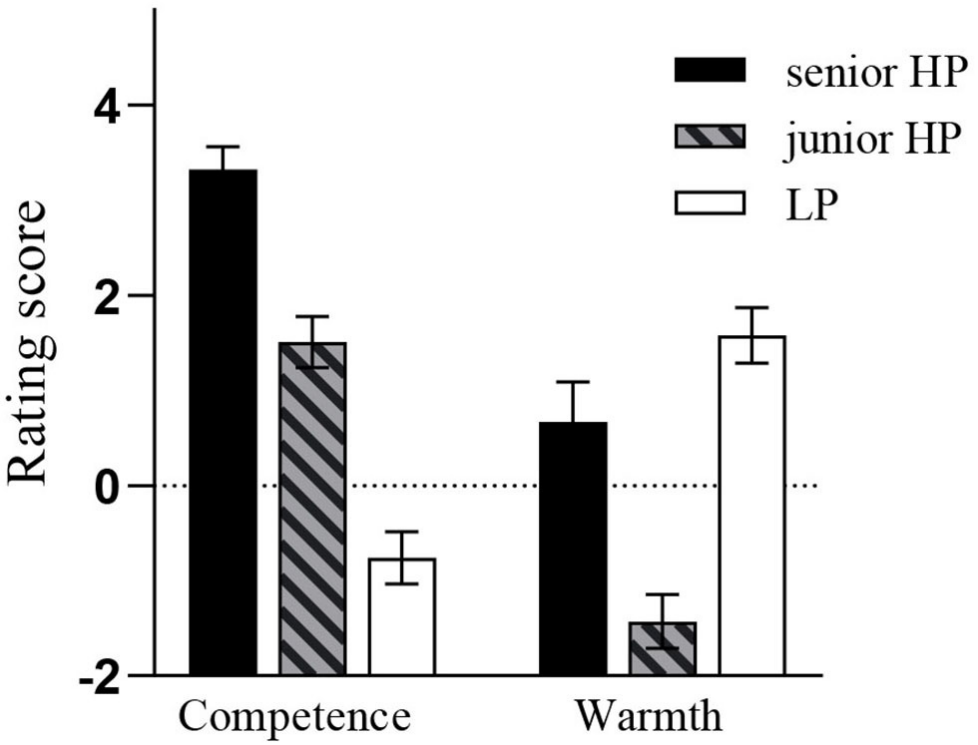
The interaction effect between stereotype dimension and target power in Study 1 was illuminated schematically. Error bar means standard error.

Again, preliminary analyses showed that gender did not produce a significant main effect or any significant interaction effects with other variables, so this variable was dropped from the formal data analyses. Then, a 3 (target power: senior HP, junior HP, LP) × 2 (stereotype dimension: competence vs. warmth) within-subjects ANOVA was conducted to examine how participants' rating scores varied with target power and stereotype dimension. This ANOVA analysis revealed a significant main effect of target power, *F*_(2, 62)_ = 25.23, *p* < 0.001, partial η^2^ = 0.45. *Post-hoc* multiple comparisons showed that the rating score toward the senior HP target was significantly higher than the other two targets, *p*s < 0.001, and no significant difference was found between the junior HP target and the LP target, *p* > 0.05 (*M*_senior_= 1.99, *M*_junior_ = 0.04, and *M*_LP_ = 0.41). The main effect of stereotype dimension was significant, *F*_(1, 31)_ = 18.38, *p* < 0.001, partial η^2^ = 0.37, as a higher score on the competence dimension than on the warmth competence (*M* = 1.36, 0.28, respectively). Importantly, the expected interaction effect between target power and stereotype dimension was significant, *F*_(2, 62)_ = 48.15, *p* < 0.001, partial η^2^ = 0.61. Simple effect analyses showed that on the competence dimension, the main effect of target power was significant, *F*_(2, 62)_ = 57.6, *p* < 0.001, partial η^2^ = 0.65. Planned comparisons revealed that participants indicated a higher score for the both senior and junior HP targets than for the LP target, *t*s > 5.99, *p*s < 0.001, ds > 1.48, and they also indicated a higher score for the senior HP target than for the junior HP target, *t*_(31)_ = 5.1, *p* < 0.001, d = 1.25. On the warmth dimension, the main effect of target power also was significant, *F*_(2, 62)_ = 22.84, *p* < 0.001, partial η^2^ = 0.42. Planned comparisons showed that participants indicated a significantly higher score for the LP target than for the junior HP target, *t*_(31)_ = 7.64, *p* < 0.001, d = 1.86, and indicated a slightly higher score for the LP target than for the senior HP target, *t*_(31)_ = 1.94, *p* = 0.06, d = 0.45. Importantly, in line with our speculation, participants indicated a significantly higher score for the senior HP target than for the junior HP target, *t*_(31)_ = 4.22, *p* < 0.001, d = 1.04. Overall, the further data analyses for the interaction between the power target and stereotype dimension suggested that participants generally tended to rate the HP targets as positive competence but negative warmth in comparison with the LP target, but the comparisons between the senior and junior HP targets showed that on both competence and warmth dimensions, participants gave more positive evaluations for the senior HP target than for the junior HP target. The schematic illustration for the interaction between power target and stereotype dimension was presented in Figure 1.

### Discussion

In Study 1, we classified power into senior HP, junior HP, and LP, then we used the trait-rating task to investigate the contents of power stereotypes. The results showed that consistent with the results of the pilot study, the HP targets, in general, were perceived as positive competence but negative warmth in comparison with the LP target. More importantly, supporting our hypothesis 2, Study 1 found that, compared with the junior HP target, participants were inclined to indicate more positive warmth evaluations for the senior HP target—the so-called MPMW effect. Indeed, not only that, but participants also gave more positive competence evaluations for the senior HP target than for the LP target. That is to say, participants indicated more positive evaluations on both competence and warmth dimensions for the senior HP target.

For the above result, we would give our interpretation, respectively, from the competence and warmth dimensions. It is a well-accepted view that competence is a basic approach to constituting power (Guinote and Vescio, 2010; Rucker et al., 2018). So, on the both intuitive and theoretical levels, it is easy to understand the phenomenon that more power was associated with higher competence. About the MPMW effect on the warmth dimension, so far there were two plausible explanations. One explanation was the Confucian explanation that we have documented. According to our reasoning, Confucianism has been the dominant philosophy in China for a long time, so most individuals in China remain high identification with the doctrines of Confucianism (Fung, 1948/2018). As a consequence, individuals under Confucian culture tend to believe that greater power represents greater social responsibility. Correspondingly, senior HP individuals are more likely to be considered to possess positive warmth traits than junior HP individuals. Under this vein, individuals' identification with Confucianism is the key factor for the appearance of the MPMW effect on HP stereotypes. Another seemingly plausible explanation was the “halo effect”. That means the MPMW effect on HP stereotypes is actually just because participants indiscriminately gave more positive evaluations toward senior HP individuals due to their greater power compared to junior HP individuals. In the following Study 2a and 2b, we would conduct empirical tests for such two explanations.

## Studies 2a and 2b

In Study 1, we classified power into senior HP, junior HP, and LP, and then applied the trait-rating task to investigate the contents of power stereotypes. Consistent with our hypothesis 2, we found the MPMW effect on HP stereotypes—more power was associated with more positive competence and warmth. Regarding this effect, there seemed to be two possible explanations: the Confucianism explanation and the “halo effect” explanation. So, the major aim of Study 2a and 2b was to further clarify which of the two explanations was appropriate for the MPMW effect observed in Study 1.

To this end, in Study 2a, we would select some participants who highly identified with Confucianism and some other participants who less identified with Confucianism to compare the power stereotypes among these participants. The underlying logic was that if participants' identification with Confucianism was the premise for the MPMW effect as we have mentioned, participants highly identifying with Confucianism would indicate more positive evaluations on both competence and warmth dimensions for senior HP target than for junior HP target, but for those individuals less identifying with Confucianism, they would indicate more positive evaluations for the senior HP individuals only on the competence dimension. By contrast, if the “halo effect” could account for the perceived more positive warmth for the senior HP target, participants should indicate more positive evaluations for senior HP individuals on both competence and warmth dimensions regardless of their identification with Confucianism. In Study 2b, we would further compare the warmth perceptions of participants from Chinese and Western cultures. Consistent with Study 2a, we speculated that Chinese participants would indicate more positive evaluations on both competence and warmth dimensions for the senior HP target than for the junior HP target while participants from Western culture would indicate more positive evaluations for the senior HP target on the competence dimension but not on the warmth dimension.

### Participants and Design

In Study 2a, there were a total of 129 participants taking part in the study on a voluntary basis (56 men, 73 women, *M*_age_ = 19.24, ranging from 17.83 to 21.75). The sample size was determined by referring to Wang and Feng (2017) work in which the research design was similar to that of the present research. For their participation, they can get 10 RMB cash or a small gift worth 10 RMB. All participants were assigned the informed consent before the formal task.

Study 2b was an online study. According to the calculation of G^*^Power 3.1 (Faul et al., 2009), the presupposed 0.1 effect size and the 0.05 significance require at least 216 participants involved in this study. Considering possible invalid data, we finally recruited 240 participants to take part in the study. All participants were recruited via Credamo, a professional data collection platform (https://www.credamo.com). Half of the participants were recruited from China-mainland (*M*_age_ = 24.83, ranging from 19.33 to 54.75, 42 men, 78 women), and the other half were recruited from America, a typically Western country (*M*_age_ = 34.54, ranging from 18.92 to 55.98; 44 men, 76 women; 115 whites, 3 blacks, 2 Asians). Chinese participants completed the Chinese version of the task and Western participants completed the English version of the task. The two versions were developed by two psychological Ph.D. students who are proficient in both Chinese and English. Before the formal task, all participants needed to learn about the academic purpose of the research, and they could continue the task only after indicating their consent to the research. For their participation, they would receive 10 RMB (for Chinese participants) or 2 USD (for Western participants).

The key design of Study 2a was a 2 (target power: senior HP vs. junior HP) × 2 (stereotype dimension: competence vs. warmth) × 2 (Confucianism identification: high vs. low) mixed-measures ANOVA with Confucianism identification as the between-subjects variable. Those participants belonging to two Confucianism-identification conditions were selected from the total of 129 participants. Additionally, with all participants, we also conducted a 2 (target power: senior HP vs. junior HP) × 2 (stereotype dimension: competence vs. warmth) within-subjects ANOVA to conceptually replicate the findings of Study 1. The design of Study 2b was a 2 (target power: senior HP vs. junior HP) × 2 (stereotype dimension: competence vs. warmth) × 2 (cultural background: Chinese culture vs. Western culture) mixed-measures ANOVA with a cultural background as the between-subjects variable.

### Materials

#### The Trait-Rating Task

The trait-rating task of Study 2a and 2b was identical to that of Study 1 except that the evaluated targets only included senior and junior HP targets. That means, the task consisted of 10 pairs of traits and two targets. For each pair of traits, participants needed to mark a number on an 11-points scale (ranging from −5 to 5) to demonstrate which trait and to what extent it was suitable to describe the target.

#### Measuring Participants' Identification With Confucianism

To our knowledge, no available scale so far can be used to assess participants' identification with Confucianism. Given that, following previous research (Gloor, 2021), a self-developed scale was applied to measure participants' identification with Confucianism. To develop this Confucianism identification scale, we firstly invited 3 undergraduates to write 5 sentences for each person which can reflect the propositions of Confucianism about how people should exert their power. Then, we invited another 10 postgraduates to evaluate on a 5-points scale to what extent each sentence was in conformity with Confucianism (1 = *extreme disconformity*, 5 = *extreme conformity*). Finally, the average score on each sentence was sorted in descending order and the top 3 sentences were selected to constitute the Confucianism identification scale. The scale was developed in Chinese and English two versions and the English version was listed here: (1) in my view, individuals with greater power should display more benevolence toward others; (2) if one person possesses social power, he/she should pay more attention to the livelihood of ordinary people; (3) those people with great social power generally have a high degree of social responsibility rather than only pursue their own enjoyment. Participants' identification with Confucianism was assessed by summarizing the score on each item of the scale. In the current research, the internal consistency of the scale was 0.84.

#### Additional Measures

In Study 2a, we developed two additional items to assess the extent to which participants have access to HP individuals. One is “in daily life, I actually have less chance to interact with senior HP individuals”, and the other is “frankly speaking, compared to junior HP individuals, I form the impressions about senior HP individuals to a greater extent based on my subjective feelings”. Considering that previous research has suggested that the differences in perceived competitiveness can evoke the differences in perceived warmth in interpersonal communication (Russell and Fiske, 2008), in Study 2b, participants were asked to report perceived competitiveness among senior and junior HP individuals.

### Procedure

Study 2a was conducted in the lab, the procedure of which was partially identical to that of Study 1. Specifically, participants arrived at the lab in a group of 8–10. Upon arrival, they were told that they would complete several impression formation tasks and then signed the informed consent. The first task was the trait-rating task which was identical to that of Study 1 except that there were only senior and junior HP targets. Following the trait-rating task, participants continued to complete the Confucianism identification scale on another page, and they also reported their interacting frequency with HP individuals. After that, we collected the necessary demographic information of participants and answered any questions about the study. Finally, we gave them a small gift of 10 RMB and guided them to leave the lab.

The data of Study 2b was collected online via the Credamo platform. Prior to the formal task, the participants needed to indicate their consent to the research. Then, they successfully completed the trait-rating task and the Confucianism identification scale. They also reported necessary demographic information. After completing all tasks, participants would receive their payments.

### Results

#### Data Analyses in Study 2a

##### The MPMW Effect on HP Individuals

One participant was dropped from data analyses because of excessive omissions. So, a total of 128 participants were included in the final data analyses. Preliminary analyses did not reveal any significant effects relevant to gender, this variable thus was not included in further data analyses. To investigate how participants' rating scores varied with target power and stereotype dimension, we conducted a 2 (target power: senior HP vs. junior HP) × 2 (stereotype dimension: competence vs. warmth) within-subjects ANOVA. The results revealed a significant main effect of power, *F*_(1, 127)_ = 76.47, *p* < 0.001, partial η^2^ = 0.38, as a higher score for the senior HP target than the junior HP target (*M* = 2.25, 0.92, respectively). The ANOVA also revealed a significant main effect of stereotype dimension, *F*_(1, 127)_ = 127.86, *p* < 0.001, partial η^2^ = 0.5, with a higher score on the competence dimension than on the warmth dimension (*M* = 2.3, 0.86, respectively). In addition, the interaction between power and stereotype dimension was significant, *F*_(1, 127)_ = 17.44, *p* < 0.001, partial η^2^ = 0.12. Simple analyses showed that the senior HP target was evaluated as more positive than the junior HP target on the both competence and warmth dimensions, but the differences between two targets were more significant on the competence dimension than on the warmth dimension, *t*_(127)competence_ = 11.93, *p* < 0.001, d = 1.33, *t*_(127)warmth_ = 3.33, *p* = 0.001, d = 0.35. The above results suggested that consistent with Study 1, Study 2a also revealed the MPMW effect on HP stereotypes.

##### The Moderating Role of Participants' Confucianism Identification for the MPMW Effect

To test whether participants' identification with Confucianism was the premise for the MPMW effect (hypothesis 3), we selected the data of those participants who display obviously high and low identities with Confucianism for further data analyses. Specifically, we first sorted the database in descending order by participants' rating scores on the Confucianism identification scale. Then, following previous research (Zou et al., 2007; Wang and Feng, 2017), we selected the top 20% cases as participants with high Confucianism identification (*M* = 14.23, *SD* = 0.86), and the bottom 20% cases as participants with low Confucianism identification (*M* = 5.82, *SD* = 1.19). An independent *T*-test showed that there was a significant difference in the rating scores on the scale between them, *t*_(50)_ = 29.12, *p* < 0.001, d = 8.23. As a consequence, a total of 52 participants were submitted into a 2 (target power: senior vs. junior) × 2 (stereotype dimension: competence vs. warmth) × 2 (Confucianism identification: high vs. low) mixed-measures ANOVA, with Confucianism identification as the between-subjects variable. Participants' average rating score in each condition was presented in Table 4.

**Table 4:** Participants' average rating score in each condition of Study 2a and 2b (*M* ± *SD*).

		**Senior HP target**	**Junior HP target**
**Study 2a**			
High Confucianism identification	Competence	3.67 ± 1.23	1.65 ± 1.51
	Warmth	2.43 ± 1.80	−0.49 ± 1.53
Low Confucianism identification	Competence	2.88 ± 1.06	1.12 ± 1.65
	Warmth	−0.08 ± 2.12	0.45 ± 2.07
**Study 2b**			
Chinese culture	Competence	3.27 ± 1.06	1.18 ± 1.56
	Warmth	0.87 ± 2.03	0.07 ± 2.29
Western culture	Competence	2.37 ± 2.06	2.15 ± 2.04
	Warmth	2.19 ± 2.01	2.21 ± 2.00

*N = 52 in Study 2a, N = 226 in Study 2b. HP, high power; LP, low power*.

The above ANOVA revealed that the main effect of target power was significant, *F*_(1, 50)_ = 41.93, *p* < 0.001, partial η^2^ = 0.46, and the subsequent *post-hoc* comparison showed that participants indicated a higher scores for the senior HP target than for the junior HP target, *t*_(51)_ = 8.73, *p* < 0.001, d = 0.97. The main effect of stereotype dimension was also significant, *F*_(1, 50)_ = 70.94, *p* < 0.001, partial η^2^ = 0.59, as a higher score on the competence dimension than on the warmth dimension, *t*_(51)_ = 8.45, *p* < 0.001, d = 1.13. Additionally, the interaction between target power and Confucianism identification was significant, *F*_(1, 50)_ = 15.17, *p* < 0.001, partial η^2^ = 0.23. However, this significant interaction was qualified by another significant interaction effect among target power, stereotype dimension, and Confucianism identification, *F*_(1, 50)_ = 17.35, *p* < 0.001, partial η^2^ = 0.26.

**Figure 2:**
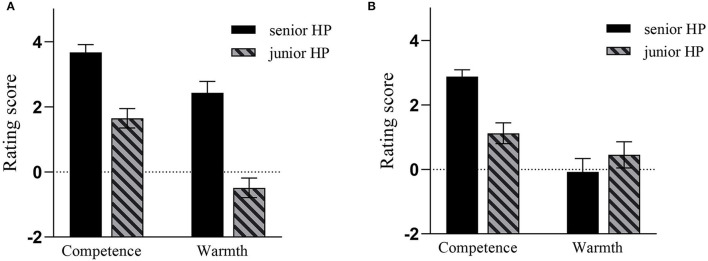
For participants with high Confucianism identification **(A)** and participants with low Confucianism identification **(B)** in Study 2a, the average rating score in each condition was presented schematically. Error bar means standard error.

For the significant effect among target power, stereotype dimension, and Confucianism identification, the subsequent simple effect analyses revealed that the interaction between target power and stereotype dimension was significant for participants who have low identification with Confucianism, *F*_(1, 25)_ = 35.46, *p* < 0.001, partial η^2^ = 0.59, whereas the interaction between them was not significant for participants who have high identification with Confucianism, *F*_(1, 25)_ = 1.86, *p* = 0.19, partial η^2^ = 0.07. Further planned analyses showed that participants with high Confucianism identification indicated a higher score for the senior HP target than for the junior HP target on both competence and warmth dimensions, *t*s > 4.93, *p*s < 0.001, but this pattern was changed for those participants with low Confucianism identification. In concrete terms, on the competence dimension, participants with low Confucianism identification indicated a significantly higher score for the senior HP target than for the junior HP target, *t*_(25)_ = 4.67, *p* < 0.001, d = 1.24 (*M* = 2.88, 1.12, respectively); however, on the warmth dimension, there were no significant differences about the scores between senior and junior HP targets, *t*_(25)_ = 1.39, *p* = 0.18, d = −0.25 (*M* = −0.08, 0.45, respectively). Simply speaking, the MPMW effect on HP stereotypes was moderated by participants' identification with Confucianism—participants with high Confucianism identification, rather than those with low Confucianism identification, were inclined to display the MPMW effect (see Figure 2).

About the item “in daily life, I actually have less chance to interact with senior HP individuals”, participants commonly alleged that they had less chance to interact with senior HP individuals”, *M* = 4.15, *SD* = 0.95 (higher value indicates less interacting experiences), and this average value was significantly greater than the median 3, *t*_(127)_ = 13.61, *p* < 0.001, d = 1.21. Correspondingly, about the item “frankly speaking, compared to junior HP individuals, I form the impressions about senior HP individuals to a greater extent based on my subjective feelings”, most participants also alleged that they formed the impressions of senior HP individuals to a greater extent based on their subjective feelings, *M* = 4.1, *SD* = 1.03, and again, this average value was significantly greater than the median 3, *t*_(127)_ = 12.06, *p* < 0.001, d = 1.07.

#### Data Analyses in Study 2b

##### The Moderating Role of Cultural Background for the MPMW Effect

A total of 14 participants were dropped from the following data analyses due to incomplete responses (1 participant) or failing to pass the attention check (5 Chinese participants, 8 Western participants). As a consequence, a total of 226 participants were included in the formal data analyses. Preliminary analyses did not reveal any significantly meaningful effects relevant to gender, so this variable was not considered in the formal data analyses. Then, we conducted a 2 (target power: senior vs. junior) × 2 (stereotype dimension: sociability vs. morality) × 2 (cultural background: Chinese culture vs. Western culture) mixed-measures ANOVA with a cultural background as the between-subjects variable. The average value in each condition was presented in Table 4. Consistent with Study 2a, the ANOVA revealed a significantly main effect of power, *F*_(1, 224)_ = 88.78, *p* < 0.001, partial η^2^ = 0.28, and a significant interaction effect between target power and stereotype dimension, *F*_(1, 224)_ = 22.45, *p* < 0.001, partial η^2^ = 0.09. However, such significant effects were qualified by a significant three-interaction effect among cultural background, target power and stereotype dimension, *F*_(1, 224)_ = 11.14, *p* = 0.001, partial η^2^ = 0.05.

**Figure 3:**
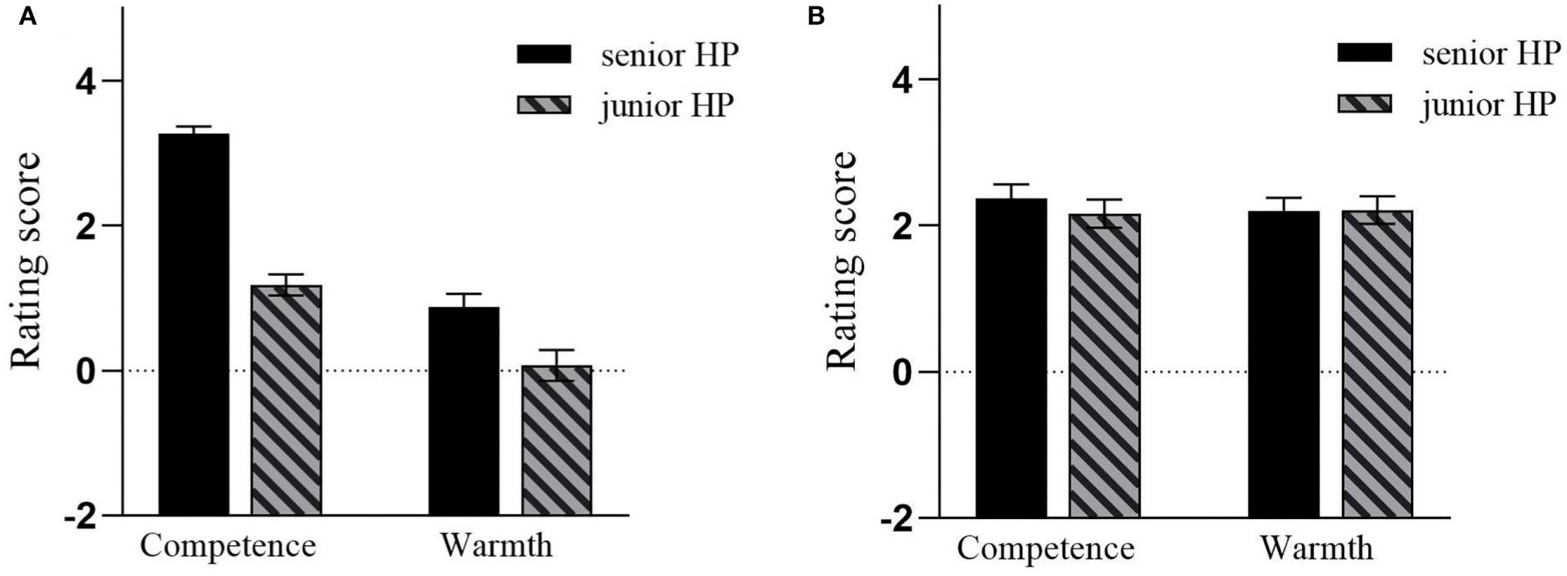
For Chinese participants **(A)** and Western participants **(B)** in Study 2b, the average rating score in each condition was presented schematically. Error bar means standard error.

To clarify the significant interaction effect among cultural background, target power and stereotype dimension, we conducted further simple effect analyses (see Figure 3). The results showed that, for Chinese participants, the effect of target power was significant, *F*_(1, 113)_ = 93.76, *p* < 0.001, partial η^2^ = 0.45, and the effect of stereotype dimension was significant, *F*_(1, 113)_ = 157.99, *p* < 0.001, partial η^2^ = 0.58. Moreover, the interaction effect between target power and stereotype dimension also was significant, *F*_(1, 113)_ = 18.22, *p* < 0.001, partial η^2^ = 0.14. For Western participants, the simple effect analysis only revealed a significant interaction effect between target power and stereotype dimension, *F*_(1, 111)_ = 5.43, *p* = 0.02, partial η^2^ = 0.05. The following simple-simple effect analyses showed that Chinese participants gave more positive evaluations for the senior HP target than for the junior HP target on both the competence and the warmth dimensions, but the significance on the competence dimension was stronger than that on the warmth dimension, *t*_(113)competence_ = 12.92, *p* < 0.001, d = 1.57, *t*_(113)warmth_ = 3.14, *p* = 0.002, d = 0.37; In contrast, Western participants only gave more positive evaluations for the senior target on the competence dimension, *t*_(111)_ = 2.6, *p* = 0.01, d = 0.11, but not on the warmth dimension, *t*_(111)_ = 0.18, *p* = 0.86.

In addition, we also compared participants' identification with Confucianism across cultures and perceived competitiveness between the senior and the junior targets. An independent *T*-test showed that Chinese participants displayed higher identification with Confucianism than Western participants (*M* = 11.20, 9.27, respectively), *t*_(224)_ = 5.53, *p* < 0.001, d = 0.74. With perceived competitiveness as the dependent variable, we performed a 2 (cultural background: Chinese culture vs. Western culture) × 2 (target power: senior vs. junior) mixed-measures ANOVA to examine whether there were differences in perceived competitiveness between senior and junior targets. This ANOVA revealed a significant effect of cultural background, *F*_(1, 224)_ = 7.38, *p* = 0.007, partial η^2^ = 0.03, with higher values for Western participants than for Chinese participants. However, the interaction effect between cultural background and target power was not significant, *F*_(1, 224)_ = 1.53, *p* = 0.22, partial η^2^ = 0.007, and the main effect of target power also was not significant, *F*_(1, 224)_ = 0.08, *p* = 0.77. Taken together, such results suggested that there were no significant differences in perceived competitiveness between the senior and the junior targets.

#### Subsidiary Analyses

In Study 2a, we conducted subsidiary analyses to exclusively explore whether the effect of target power on participants' warmth perception varied with specific traits. Specifically, following previous research (Leach et al., 2007), we further classified five warmth traits into sociability and morality two sub-dimensions. To assess the feasibility of this classification, a CFA was conducted with participants' ratings on the warmth traits. The results showed a good mode fitness, χ^2^*/df* = 1.18, *p* = 0.32, *CFI* = 0.99, *TLI* = 0.99, *SRMR* = 0.01, *RMSEA* = 0.04, suggesting that our classification can be acceptable. Then, we performed a 2 (target power: senior vs. junior) × 2 (warmth sub-dimension: sociability vs. morality) within-subjects ANOVA with all participants in Study 2a. The results showed a significant main effect of target power, *F*_(1, 127)_ = 8.40, *p* = 0.004, partial η^2^ = 0.06, and a significant interaction effect between target power and warmth sub-dimension, *F*_(1, 127)_ = 23.21, *p* < 0.001, partial η^2^ = 0.15. The following simple effect analyses showed that participants indicated a significantly higher score for the senior HP target than for the junior HP target on the sociability sub-dimension (*M* = 1.51, 0.32, respectively), *t*_(127)_ = 4.66, *p* < 0.001, d = 0.52, but there were no significant differences between two targets on the morality sub-dimension (*M* = 0.87, 0.7, respectively), *t*_(127)_ = 0.67, *p* = 0.51, d = 0.07.

### Discussion

Consistent with the results of Study 1, Study 2a also showed that compared to the junior HP target, the senior HP target tended to be perceived as more positive on the warmth dimension, thus providing the collaborating evidence for our hypothesis 2. For the MPMW effect on the warmth dimension, as we have mentioned in Study1, the Confucianism explanation and the “halo effect” explanation were both plausible explanations. However, in Study 2a, when we intentionally selected some participants displaying high/low identification with Confucianism, we found that participants with high Confucianism identification indicated more positive evaluations for the senior HP target on both competence and warmth dimensions, but for participants with low Confucianism identification, they gave the senior HP target more positive evaluations only on the competence dimension. Although the “halo effect” hypothesis could explain the result that participants with high Confucianism identification gave the senior HP target more positive evaluations on both competence and warmth dimensions, it could not explain why participants with low Confucianism identification gave the senior HP target more positive evaluations only on the competence dimension. Thus, the “halo effect” hypothesis can be basically eliminated from our research. Thus, Study 2a provided the initial support for our Confucianism explanation.

In Study 2b, we conducted a cross-cultural study and found that the MPMW effect emerged for Chinese participants but not for Western participants. Moreover, this perceived warmth difference between senior and junior HP targets could not be explained by the perceived competitiveness between senior and junior HP targets. Additionally, our subsidiary analyses showed that when those warmth traits were distinguished as sociability and morality, participants gave more positive evaluations for the senior HP target than for the junior HP target on the sociability sub-dimension, but not on the morality sub-dimension. This result suggested that participants' relatively negative warmth perception about the junior HP target may stem from their negative expectation about the social agreeableness of junior HP individuals, which to some extent provided additional support for our Confucianism explanation. Taken together, by measuring participants' identification with Confucianism on the individual level and conducting a cross-cultural study, the results of Study 2a and 2b consistently demonstrated that participants' identification with Confucianism was the premise for the MPMW effect.

## Study 3

In Study 2a, by deliberately selecting some participants with high/low Confucianism identification under Chinese culture, we found that the MPMW effect emerged for participants with high Confucianism identification but not for participants with low Confucianism identification. In Study 2b, by conducting a cross-cultural study including Chinese and Western participants, we found that the MPMW effect emerged for Chinese participants but not for Western participants. The results of Study 2a and 2b consistently provided explicit evidence for our hypothesis 3 that participants' Confucianism identification was the premise for the MPMW effect on HP stereotypes. In Study 3, we aimed to provide implicit evidence for the role of Confucianism identification in the MPMW effect. Specifically, in Study 3, we would apply the IAT to measure participants' power stereotypes on the implicit level. The IAT, originally developed by Greenwald et al. (1998), has been widely used to measure the implicit association strength of multiple stereotypes (Lai and Wilso, 2020). Compared to explicit measures, the IAT was considered to be less susceptible to social desirability effects, and thus more likely to detect individuals' internal “unshaped” beliefs (Nosek et al., 2011; Lai and Wilso, 2020). Thus, in the current research, the IAT was used to further confirm the MPMW effect on HP stereotypes and specify the condition for the emergence of the phenomenon.

In Study 3, we sought to use the priming technique to temporarily reduce participants' identification with Confucianism, and then examine the role of Confucianism in the emergence of the MPMW effect. Specifically, we randomly assigned participants to the control and the counter-Confucianism conditions. Under the control conditions, participants firstly read two paragraphs irrelevant to Confucianism and then completed the IAT. Under the counter-Confucianism condition, participants firstly read two paragraphs conveying the gist in opposition to Confucianism and then completed the IAT. More detailed descriptions about the IAT were provided in the Section Materials.

In the IAT, we defined senior and junior HP labels as target dimensions and defined positive and negative warmth traits as attribute dimensions. Because previous explicit results suggested that on the warmth dimension, participants gave senior HP individuals more positive evaluations than junior HP individuals, in the IAT, the combinations of “senior HP—positive warmth” and “junior HP—negative warmth” were defined as the compatible responses, and the combinations of “senior HP—negative warmth” and “junior HP—positive warmth” were defined as the incompatible responses. Following previous research (Greenwald et al., 2003), the calculated D value was the indicator of implicit association strength of HP stereotypes and greater D values represented stronger strength of HP stereotypes. According to our reasoning, we speculated that when the counter-Confucianism information was presented, the D value would be significantly lower under the counter-Confucianism condition than that under the control condition (no counter-Confucianism information).

### Participants

According to the calculation of G^*^Power 3.1 (Faul et al., 2009), a presupposed 0.8 effect size and 0.05 significance required at least 84 participants to participate in Study 3. Given this, on a voluntary basis, we finally recruited 85 undergraduates to take part in the study (27 men, 58 women, *M*_age_ = 21.03, ranging from 19 to 23.5). They all assigned the informed consent before the formal tasks.

### Materials

#### The Reading-Comprehension Task

In Study 3, we used a so-called “reading-comprehension task” to present the counter-Confucianism information to participants. Concretely, in this so-called reading-comprehension task, approximately half of the participants were asked to read two passages that mainly told readers that although HP individuals under Confucian culture, including senior and junior HP individuals, alleged their social responsibility and benevolence for the whole society, they actually were likely to serve themselves by exerting their power. To increase the credibility of such counter-Confucianism materials, we provided some theoretical or practical evidence for our argument. To further reinforce the priming effect of the counter-Confucianism materials, all participants were required to extract and write the gist of each paragraph. Previous literature concerning gender stereotypes suggests that presenting the counter-stereotype materials to participants can effectively elevate the accessibility of stereotype-inconsistent information and reduce the strength of gender stereotypes (Dasgupta and Asgari, 2004). One of the two passages was translated into the English version and presented here as an example.

*In China, when ordinary people suffer from unfair treatments from junior HP individuals, they prefer to ask for the assistance from senior HP individuals. As an example, in ancient China, those people who dare to tell their suffering to the then emperor in a face-to-face way, even risking their lives, have been legends in the public mind. We can find many such legends in a variety of literary works. Indeed, this interesting phenomenon may root in the Confucian culture. Specifically, since the Han Dynasty, Confucianism has been the dominant culture in China, which proposes that a “qualified” HP person should have a high degree of social responsibility and devote his life to serving for the whole society. Due to this chronic influence of Confucianism, most Chinese people are willing to believe that more power is associated with more social responsibility. Thus, compared to junior HP individuals, senior HP individuals are considered to be more likely to help ordinary people solve problems. However, what about the facts? Psychological research indicates that power can increase psychological distance from others. That means, the more power one person holds, the less motivation to interact with others. Correspondingly, it actually is a difficult thing for HP individuals to pay much attention to others, especially to ordinary people. As a consequence, although Confucianism alleges that more power represents more social power and most people also believe that that's the case, unfortunately, once holding power, regardless of senior or junior HP individuals, they may do not have enough motivation to actively help ordinary people solve problems*.

It should be noticed that the counter-Confucianism materials did not explicitly contain descriptions literally at odds with HP stereotypes (e.g., senior HP individuals commonly are hypocritical). Rather, the counter-Confucianism materials conveyed the implications contrary to the doctrines of Confucianism. That is, once holding power, regardless of senior or junior HP individuals, they will be less motivated to pay much attention to others; in contrast, they are more likely to afford their own needs by exerting their power. Thus, in the counter-Confucianism condition, we sought to reduce the implicit association strength of HP stereotypes by temporarily reducing participants' identification with Confucianism. In the control condition, the other half of the participants read two neutral materials which were both matched with the experimental materials in length and readability. One of the two passages introduced some UFO incidents in the past several decades, and the other introduced the formation and evolution of Antarctic glaciers. Participants in the experimental and control conditions were both instructed to complete the reading-comprehension task within 10 min.

#### The IAT Task

In Study 3, the classic IAT was employed to assess the strength of HP stereotypes across two conditions. In the IAT task, the target dimension consisted of 8 HP labels (4 senior HP and 4 junior HP) and the attribute dimension consisted of 16 warmth traits (8 positive traits and 8 negative traits). All target and attribute words were selected from previous relevant research and listed in Table 5 (Zhang et al., 2013; Wang et al., 2017).

**Table 5:** HP labels on the target dimension (senior vs. junior) and warmth traits (positive vs. negative) on the attribute dimension in the IAT of Study 3.

**Target dimension**		**Attribute dimension**	
**Senior HP**	**Junior HP**	**Positive warmth**	**Negative warmth**
Minister	Section Chief	Friendly	Unfriendly
Board Chairman	Workshop Manager	Upright	Cunning
Director General	Community Director	Genuine	Hypocritical
Senior officials	Village Secretary	Enthusiastic	Indifferent
		Modest	Supercilious
		Easy-going	Domineering
		Amiable	Rigid
		Altruistic	Self-serving

*IAT, implicit association task; HP, high power; LP, low power*.

The IAT task was run by E-prime 3.0 on the computer. The classic IAT included 7 parts. At the beginning of each part, a task instruction was presented on the computer screen until participants pressed the space key, and then, participants needed to classify each stimulus by pressing the *E* or *I* key. In part 1, participants were instructed to classify 24 warmth traits into the “positive” or “negative” category. The 24 warmth traits consisted of all 16 warmth traits and the selected 8 traits from them in a pseudo-random way (4 positive warmth traits and 4 negative warmth traits). They needed to press the *E* key if a positive word was presented and they needed to press the *I* key if a negative word was presented. When they pressed the wrong key, the feedback “wrong response (in Chinese)” would be presented on the screen. Similarly, participants needed to classify 24 target labels into the “senior HP” or “junior HP” category (8 HP labels were presented three times) in part 2. They needed to press the *E* key for senior HP labels and press the *I* key for junior HP labels. Again, the feedback “wrong response” would be presented on the screen upon participants pressing an incorrect key. The main purpose of parts 1 and 2 was to make participants familiar with experimental stimuli and correct key-press.

Part 3 and 4 were compatible combination-response parts. As we have mentioned above, we proposed that on the relative level, participants tended to associate senior HP labels with positive warmth traits and associate with junior HP labels with negative warmth traits. So, in part 3, participants needed to press the *E* key when senior HP labels or positive warmth traits were presented on the screen, and they needed to press the *I* key when junior HP labels or negative warmth words were presented on the screen. The task demand of part 4 was identical to that of part 3 except for the trial quantity—part 3 consisted of 24 trials and Block 4 consisted of 48 trials.

In part 5, participants made the key-press contrary to part 1. That is, they needed to press the *E* key for negative warmth traits and press the *I* key for the positive warmth traits. To make participants fully familiar with this new key-press, part 5 consisted of 48 trials.

Parts 6 and 7 were incompatible combination-response parts in which participants needed to press the *E* key for senior HP labels or negative warmth traits and press the *I* key for junior HP labels or positive warmth traits. Similar to parts 3 and 4, part 6 consisted of 24 trials and part 7 consisted of 48 trials. In general, participants took about 15–17 min to complete the whole IAT task.

##### The Confucianism Identification Scale

In Study 3, the Confucianism identification scale was used to check the effectiveness of the counter-Confucianism priming. After completing the IAT task, participants needed to fill out the scale to compare whether there were significant differences in participants' identification with Confucianism between the counter-Confucianism and control conditions.

### Procedure

Participants took part in the study in a group of 6 and they arrived at the lab at the appointed time. Upon arrival, they were told that they would complete two (ostensibly) unrelated tasks for the purpose of saving time. Firstly, they completed the reading-comprehension task in which approximately half of the participants read two passages containing the counter-Confucianism information and the other half read two paragraphs unrelated to Confucianism. After reading each paragraph, participants needed to write its gist in one sentence. Following the reading-comprehension task, participants continued to complete the IAT task. Then, participants filled out the Confucianism identification scale, and also provided necessary demographic information. Finally, they were thanked, debriefed, and guided to leave the lab. The whole task lasted about 25–30 min.

### Results

The data of 2 participants (1 man, 1 woman) failed to be collected successively due to an unknown software error. Thus, a total of 83 participants were included in the final data analyses. Preliminary analyses showed that gender did not produce any significant main or interaction effects, so this variable was dropped from the following data analyses.

#### The Check for the Manipulation of the Counter-Confucianism Priming

During the debriefing session, no participant expressed suspicion about the authenticity of the presented stimulus in the reading-comprehension task. The effectiveness of the counter-Confucianism was estimated by summarizing each participant's score on three items of the Confucianism identification scale. The subsequent independent *T*-test revealed that participants in the counter-Confucianism condition reported a significantly lower score on the Confucianism identification scale than participants in the control condition, *t*_(81)_ = 6.55, *p* < 0.001, d = 1.44 (*M* = 8.49, 11.95, respectively), thus demonstrating the effectiveness of our manipulation. The presentation of the counter-Confucianism stimuli temporarily reduced the accessibility of Confucianism, and participants in the counter-Confucianism condition thus showed relatively lower identification with Confucianism.

#### The IAT Task

Following the recommendation by Greenwald et al. (2003), the D value of each participant was calculated as the indicator of the strength of HP stereotypes. Two one-sample *T*-tests revealed that the D values in the counter-Confucianism and control conditions were both significantly > 0, *t*s > 12.36, *p*s < 0.001, suggesting that participants in both conditions showed the MPMW effect on HP stereotypes. Then, we conducted an independent *T*-test to compare the D values between the counter-Confucianism and control conditions. The results showed that the D value in the counter-Confucianism condition was significantly smaller than the D value in the control condition, *t*_(81)_ = 3.27, *p* = 0.002, d = 0.72 (*M* = 0.66, 0.9, respectively), suggesting that the MPMW effect was significantly attenuated when presenting the counter-Confucianism materials.

### Discussion

To further confirm the role of Confucianism in the MPMW effect, in Study 3, we used the priming technique to manipulate the accessibility of Confucianism and then applied the IAT task to compare the strength of the MPMW effect between the counter-Confucianism and control conditions. Consistent with our speculation, the D value in the counter-Confucianism condition was significantly lower than that in the control condition, suggesting the weaker stereotype strength in the counter-Confucianism condition. From the perspective of the accessible theory (Förster and Liberman, 2007; Higgins, 2012), when the counter-Confucianism stimuli were presented to participants, the temporarily accessible counter-Confucianism knowledge reduced participants' reliance on the chronically accessible Confucianism. As a reflection, the D value in the counter-Confucianism condition was significantly reduced than that in the control condition. Notably, despite significant differences in the D values between the counter-Confucianism and control conditions, the D values in both conditions were both significantly larger than zero. This finding suggested that the presentation of the counter-Confucianism information could reduce the MPMW effect, but it cannot eliminate the effect. Indeed, our finding was consistent with previous stereotype literature, which consistently demonstrated that although stereotypes display quite a flexibility and function, stereotypes are less likely to be completely eliminated (Fiske and Neuberg, 1990; Ellemers, 2018).

In Study 2, by measuring participants' identification with Confucianism on the individual level (2a) and selecting participants from Chinese and Western cultures (2b), we found that participants for whom Confucianism was chronically accessible knowledge, rather than participants for whom Confucianism was less accessible knowledge, tended to display the MPMW effect about HP stereotypes. In Study 3, we further found that when the accessibility of the counter-Confucianism information was temporarily elevated, participants showed a weaker MPMW effect in the IAT task. Thus, studies 2 and 3 provided compelling evidence for our hypothesis that Confucianism was a key premise for the MPMW effect on HP stereotypes—only when Confucianism is accessible in a specific situation, do individuals tend to associate more power with more warmth with HP stereotypes.

## General Discussion

In the current research, we classified power in a relatively continuous way and conducted 5 studies to mainly investigate the MPMW effect on HP stereotypes and the condition for the emergence of the effect. By using the trait-rating task, the pilot study replicated previous research and revealed that in comparison with LP individuals, HP individuals tended to be perceived as positive competence but negative warmth (Russell and Fiske, 2008; Fragale et al., 2009; Zhang et al., 2013, 2015; Wang et al., 2017). The following Study 1 demonstrated that when HP was further divided into senior and junior HP, participants gave more positive evaluations on both competence and warmth dimensions for senior HP individuals than for junior HP individuals, thus providing initial evidence for the MPMW effect on HP stereotypes—more power tended to be associated with more perceived warmth about HP individuals. By enacting the trait-rating task in a larger sample, Study 2a further confirmed the MPMW effect on HP stereotypes. More importantly, Study revealed that the MPMW effect was more likely to emerge for participants with high Confucianism identification (2a) or participants from Confucianism-dominant culture (2b), thus suggesting that participants' identification with Confucianism may be a premise for the emergence of the MPMW effect. In Study 3, by presenting the Counter-Confucianism stimuli to temporarily reduce the accessibility of Confucianism, we found that the D value in the IAT under the Counter-Confucianism condition was significantly lower than that under the control condition. This result indicated that because of the decline of the Confucianism accessibility, the strength of the MPMW effect had been significantly reduced, thus providing convincing evidence for our hypothesis that participants' identification with Confucianism was the premise for the existence of the MPMW effect.

### The MPMW Effect and Confucianism

Recently, Anicich et al. (2020) have pointed out that “an unstated premise in the social power literature is that an individual either has or lacks power”, and a large body of literature also has exclusively focused on the static comparison between HP and LP individuals, thus to a great extent ignoring the dynamic nature of power (Anicich et al., 2020). Following the proposition by Anicich et al. (2020), the present research not only investigated the contents of power stereotypes about HP and LP individuals by adopting a simply dualistic classification approach (HP/LP) but also further divided HP into senior and junior HP by adopting a relatively continuous classification approach. The results showed that when adopting a dualistic classification approach for power, the current research kept consistent with previous research—participants tended to evaluate LP individuals as negative competent but positive warmth and evaluate HP individuals as positive competent but negative warmth. In a simple word, elevated power was considered to be associated with more negative warmth. However, when HP was subdivided into senior HP and junior HP, we found that the evaluation of senior HP individuals' warmth did not linearly become more negative along with the elevation of power, and in contrast, senior HP individuals were considered to display more positive warmth than junior HP individuals. Regarding this so-called MPMW effect on HP individuals, our research revealed that participants' identification with Confucianism was a key factor in its existence. When participants had less identification with Confucianism on the individual level or temporarily had relatively less identification with Confucianism due to experimental manipulation, the MPMW effect had been significantly reduced, even eliminated.

Additionally, we found that when comparing senior and junior HP individuals with LP individuals, participants, in general, gave more positive warmth evaluations for LP individuals than for HP individuals (including senior and junior HP individuals). In other words, the current research did not challenge previous literature which consistently demonstrates that LP individuals are considered to display more positive warmth than HP individuals (Cuddy et al., 2008; Russell and Fiske, 2008; Fragale et al., 2009; Zhang et al., 2013; Wang et al., 2017). That implies, under Confucian culture, although explicitly differentiating senior and junior HP individuals could evoke participants' positive expectations toward senior HP individuals, this could not completely counteract their negative warmth evaluations toward HP individuals on the whole. To some extent, the result also suggests that Confucianism and individuals' real experiences may both contribute to the formation of power stereotypes under Chinese background, however, those real experiences may carry more weight in power stereotype formation. From a special perspective, the key role of Confucianism in the MPMW effect actually reminds us that we should be extremely cautious in assessing the representativeness and generality of the MPMW effect. At least according to our results, the MPMW effect may only exist among HP stereotypes under Confucian culture, and this conclusion still needs to be further assessed by conducting cross-cultural research.

### Implications for Power Stereotype Research

At first glance, our finding that senior HP individuals were perceived as warmer than junior HP individuals was at odds with the theoretical propositions of the SCM (Fiske et al., 2002; Cuddy et al., 2008). According to the SCM, individuals tend to form ambivalent stereotypes about out-group members—a positive evaluation on one dimension and a negative evaluation on the other. Moreover, the SCM posits that the elevation of competition and status often means more positive evaluation on the competence dimension but more negative evaluations on the warmth dimension. So, according to this proposition, senior HP individuals seemingly should be perceived as more negative on the warmth dimension compared to junior HP individuals. We proposed that this superficial contradiction could be accounted for by the term “reference group” of the SCM (Cuddy et al., 2008, 2009). Specifically, the SCM defines the reference groups as in-groups to which perceivers belong or societal prototype groups which theoretically serve as normative standards for social comparison or social aspiration (Cuddy et al., 2009). According to this definition, senior HP individuals could be classified into the latter case, because Study 2a revealed that the great majority of participants reported that they actually had little chance to get access to senior HP individuals and they may form impressions about senior HP individuals mainly based on the construction of Confucianism. In this sense, the MPMW effect on senior HP individuals actually reflects participants' expectation about HP individuals. In other words, senior HP individuals may be regarded as the reference group and the MPMW effect on them reflect the expectation of the whole Chinese society about how HP individuals should exert social power. Thus, our finding in essence is consistent with the propositions of the SCM.

On a broad level, the current research suggested that power stereotypes could serve to maintain the stability of social hierarchy, thus indicating the social function of power stereotypes (Ryan, 2002). As is well known, stereotypes function as a double-edged sword. On one hand, stereotypes can bias individuals' cognition and behavior, and even lead to particular kinds of discrimination and conflict in society (Bordalo et al., 2016). However, on the other hand, stereotypes also help us effectively navigate the social world. Specifically, on the individual level, the cognitive miser hypothesis postulates that in an environment that contains too much information, individuals apply specific stereotypes to simplify their information processing and enhance their processing efficiency (Brewer, 1988; Fiske and Neuberg, 1990). In addition to enhancing the efficiency of processing information, according to the “kernel of truth” hypothesis which contends that stereotypes may represent the over-amplified but the real existing differences between groups, stereotypes also provide a quick and intuitive assessment for us when confronting strangers or groups (Oakes et al., 1994). On the social level, some researchers have proposed that stereotypes to some extent reflect people's propensity to follow the social rules within a particular culture, and stereotypes can satisfy people's this need by serving to maintain the legitimacy and stability of social hierarchy (Tajfel, 1981; Leyens and Fiske, 1994). Supporting the view of the social function of stereotypes, our results suggest that possessing power stereotypes to some extent serves for maintaining the stability of social hierarchy. For example, when ordinary people realize that they have received unfair treatment from junior officials of the government (e.g., village head), they generally do not immediately take action against the government. Rather, they often choose to express their voices toward senior officials of the government (e.g., county head), which is called “applying for an audience with the higher authorities to appeal for help” in Chinese society. According to our results, the logic underlying this phenomenon may be that most ordinary people believe that those leaders with greater power can handle matters in line with the doctrines of Confucianism and treat them fairly and kindly. In this sense, the social function of power stereotypes is similar to the function of the system justification theory (SJT), which posits that people generally perceive the prevailing social system as fair and legitimate, and are willing to maintain or bolster the stability of the whole society (Jost and Banaji, 1994; Kay and Jost, 2003).

### Limitations and Future Work

Several limitations still existed in the current research. Firstly, in Study 2a, we found that participants indicated slightly more positive evaluations on the warmth dimension for the LP target than for the senior HP target. Considering that our main concern was the comparison of the perceived warmth between senior and junior HP targets, we thus did not further compare the perceived warmth between senior HP and LP targets. Given that, we actually could not draw a definite conclusion about the warmth comparison between senior HP and LP stereotypes under Confucian culture. This issue deserves further investigation. Also in Study 2a, our additional analyses showed that compared to junior HP targets, participants displayed more positive perceptions of senior HP targets on the sociability sub-dimension rather than on the morality sub-dimension. On the hand, this finding provided additional support for our Confucian explanation. However, on the other hand, it also reminds us that dividing traits into competence and warmth dimensions may be an oversimplified classification, and in future research, a more subtle classification of the trait level will help us have a deeper understanding about the MPMW effect. Thirdly, in Study 3, we found that the implicit evaluations toward senior HP targets on the warmth dimension became less positive when participants were presented with negative exemplars about senior HP individuals, but it is still certain whether the exposure to positive exemplars also could improve the evaluations toward junior HP individuals. In daily life, as we have described, most people actually have a high likelihood to interact with junior HP individuals rather than senior HP individuals. So, improving the evaluations of junior HP individuals may carry important practical implications for the stability of social order (Magee and Galinsky, 2008), and we should make a confirmation about this issue. Finally, due to lacking an available scale to assess individuals' identification with Confucianism, we developed the Confucianism scale containing three items. Although the scale displayed good internal consistency reliability in the current research, its suitability under Confucian culture should be completely assessed in a larger sample. Additionally, considering that the current research was the first time to reveal the MPMW effect on senior and junior HP individuals, we should be cautious about the generalizability of the effect, and more work still needed to be done to solve this issue.

## Conclusions

By employing the trait-rating task and the IAT task, the current research found that under Chinese culture, although participants tended to associate more power with less warmth when comparing HP individuals with LP individuals, they associated more power with more warmth when comparing senior HP with junior HP individuals (the MPMW effect). By measuring/manipulating the accessibility of Confucianism, the current research further specified that participants' identification with Confucianism was the premise for the existence of the MPMW effect. The present research for the first time demonstrates that the contents of power stereotypes may partially display culture-specific characteristics under Confucian culture. Additionally, the continuous perspective on power classification in our research provides a novel insight for future power research.

## Data Availability Statement

The raw data supporting the conclusions of this article will be made available by the authors, without undue reservation.

## Ethics Statement

The studies involving human participants were reviewed and approved by Taishan University. The patients/participants provided their written informed consent to participate in this study.

## Author Contributions

FY designed the current research. ML wrote and revised the manuscript. YH collected the data involved in the research and conducted data analyses. All authors contributed to the article and approved the submitted version.

## Funding

The research was supported by a project from Taishan University (No. 5013200223).

## Conflict of Interest

The authors declare that the research was conducted in the absence of any commercial or financial relationships that could be construed as a potential conflict of interest.

## Publisher's Note

All claims expressed in this article are solely those of the authors and do not necessarily represent those of their affiliated organizations, or those of the publisher, the editors and the reviewers. Any product that may be evaluated in this article, or claim that may be made by its manufacturer, is not guaranteed or endorsed by the publisher.
